# Reducing Parental Uncertainty Around Childhood Cancer: Implementation Decisions and Design Trade-Offs in Developing an Electronic Health Record-Linked Mobile App

**DOI:** 10.2196/resprot.7523

**Published:** 2017-06-26

**Authors:** Keith Marsolo, William Shuman, Jeremy Nix, Caroline F Morrison, Larry L Mullins, Ahna LH Pai

**Affiliations:** ^1^ Division of Biomedical Informatics Cincinnati Children's Hospital Medical Center Cincinnati, OH United States; ^2^ Department of Pediatrics University of Cincinnati College of Medicine Cincinnati, OH United States; ^3^ Division of Behavioral Medicine and Clinical Psychology Cincinnati Children's Hospital Medical Center Cincinnati, OH United States; ^4^ Department of Psychology Oklahoma State University Stillwater, OK United States

**Keywords:** electronic health records, mobile apps, uncertainty, ethnographic design

## Abstract

**Background:**

Parents of children newly diagnosed with cancer are confronted with multiple stressors that place them at risk for significant psychological distress. One strategy that has been shown to help reduce uncertainty is the provision of basic information; however, families of newly diagnosed cancer patients are often bombarded with educational material. Technology has the potential to help families manage their informational needs and move towards normalization.

**Objective:**

The aim of this study was to create a mobile app that pulls together data from both the electronic health record (EHR) and vetted external information resources to provide tailored information to parents of newly diagnosed children as one method to reduce the uncertainty around their child’s illness. This app was developed to be used by families in a National Institutes of Health (NIH)-funded randomized controlled trial (RCT) aimed at decreasing uncertainty and the subsequent psychological distress.

**Methods:**

A 2-phase qualitative study was conducted to elicit the features and content of the mobile app based on the needs and experience of parents of children newly diagnosed with cancer and their providers. Example functions include the ability to view laboratory results, look up appointments, and to access educational material. Educational material was obtained from databases maintained by the National Cancer Institute (NCI) as well as from groups like the Children’s Oncology Group (COG) and care teams within Cincinnati Children’s Hospital Medical Center (CCHMC). The use of EHR-based Web services was explored to allow data like laboratory results to be retrieved in real-time.

**Results:**

The ethnographic design process resulted in a framework that divided the content of the mobile app into the following 4 sections: (1) information about the patient’s current treatment and other data from the EHR; (2) educational background material; (3) a calendar to view upcoming appointments at their medical center; and (4) a section where participants in the RCT document the study data. Integration with the NCI databases was straightforward; however, accessing the EHR Web services posed a challenge, though the roadblocks were not technical in nature. The lack of a formal, end-to-end institutional process for requesting Web service access and a mechanism to shepherd the request through all stages of implementation proved to be the biggest barrier.

**Conclusions:**

We successfully deployed a mobile app with a custom user interface that can integrate with the EHR to retrieve laboratory results and appointment information using vendor-provided Web services. Developers should expect to face hurdles when integrating with the EHR, but many of them can be addressed with frequent communication and thorough documentation. Executive sponsorship is also a key factor for success.

**Trial Registration:**

ClinicalTrials.gov NCT02505165; https://clinicaltrials.gov/ct2/show/NCT02505165 (Archived by WebCite at http://www.Webcitation.org/6r9ZSUgoT)

## Introduction

Parents of children newly diagnosed with cancer are confronted with multiple stressors that place them at risk for significant psychological distress. In a short span of time, they are expected to become familiar with medical terminology, learn and understand treatment protocols, manage complicated medication regimens and support their child through the associated side effects, communicate with their child’s care team, and attend numerous testing and medical appointments. Parents must do all of this while advocating and providing emotional support for their child as well as continue to manage the ongoing responsibilities of home, career, and in many cases, their other children [1]. For many parents, the sudden and intensive demands generate feelings of uncertainty and can lead to symptoms of anxiety and depression, impaired cognition, and disrupted sleep [2,3]. Not surprisingly, parental distress can negatively impact a child’s well-being as well. Therefore, addressing the needs of parents is important for the health and well-being of both parent and child during this difficult peri-diagnostic period [1,4-6].

One strategy that has been shown to reduce uncertainty is the provision of basic information [7]; however, families of newly diagnosed cancer patients are often bombarded with educational material. At the time of diagnosis, well-meaning medical teams typically provide every family with a large binder that contains hundreds of pages of paper documentation. Given to the family all at once, much of the binder content may not be applicable to their child and is often only relevant during a particular phase of treatment. For a number of parents, the binder, provided with the intent to decrease uncertainty, may have the opposite effect. Still other parents may avoid the binder altogether and never open it. In contrast, technology has the potential to help families manage their informational needs and move towards normalization of their lives [8]. As the “5 Rights” framework, established for decision support, seeks to deliver the right information to the right person(s), using the right format, in the right channel, at the right point in the workflow [9], the same 5 rights can be applied in helping deliver the most relevant information to families of children with cancer at the proper time.

We sought to create a mobile app that pulls together information from both the electronic health record (EHR) and external and vetted information resources, in order to provide tailored information to parents of newly diagnosed children as one method to reduce the uncertainty around their child’s illness. To develop an optimal app interface, an interdisciplinary team conducted a user-centered design process to define and prioritize the content and features. The application was developed to be used by families in a National Institutes of Health (NIH)-funded randomized controlled trial (RCT) aimed at decreasing uncertainty and the subsequent psychological distress. This RCT is currently underway and recruiting at Cincinnati Children’s Hospital Medical Center (CCHMC) and the University of Oklahoma Health Sciences Center (OUHSC). This paper describes the implementation decisions that occurred in translating the output of the design process into a functioning mobile app along with best practices and recommendations for others embarking upon similar projects.

## Methods

### Trial Design

The proposed trial seeks to recruit up to 300 participants over a 3-year period, with an additional year for follow-up and analysis. Participants are recruited from patients at CCHMC or OUHSC who are under the age of 18 and who are newly diagnosed with leukemia, lymphoma, or a malignant tumor (2 to 12 weeks post diagnosis). The parents of the patients are also recruited. Participants are randomized into one of the following 2 interventions: (1) Illness Management and Parental Adjustment to Cancer Treatment (IMPACT), or (2) education and support only (ESO), which serves as the control. Parents receiving the IMPACT intervention will participate in 6 sessions where they receive feedback on ways to manage their uncertainty, with the first 3 sessions focused on uncertainty prevention and the last 3 on responses for situations in which uncertainty cannot be prevented or avoided. In addition, parents have access to the mobile app, which will increase their access to knowledge about their child’s illness and treatments. Those in the ESO (control) group will participate in 6 sessions, where they will receive general support as well as education on cancer etiology, medical treatments, potential short- and long-term effects of treatment, and other resources that are often helpful to parents of children with cancer. The hypothesis is that IMPACT will teach parents about uncertainty prevention and management through the use of medically-specific communication, information management, and problem-solving skills. Parents and children are expected to complete online measurements at baseline and the 1-week and 3-, 6- and 12-month follow-up appointments. The primary outcome measure is psychosocial functioning as assessed by the Global Severity Index of Symptom Checklist-90-Revised [10]. The secondary outcome measure is the post-traumatic stress symptoms score as assessed by the Impact of Events Scale-Revised [11]. Potential mediators of the treatment effects, including levels of uncertainty and use of the mobile app, will also be examined.

### Ethnographic Design Process

A 2-phase qualitative study was conducted to elicit the features and content of the mobile app based on the needs and experience of parents of children newly diagnosed with cancer and their providers [12]. The first phase consisted of semi-structured interviews with children with cancer and their parents. In the second phase, caregivers and healthcare providers were asked to identify and rank a series of app functions that were derived from the results of the interviews in phase 1. Example functions include the ability to view laboratory results, look up appointments, and to quickly access educational material through a search function. The output of this second phase was then converted into a series of wireframes. These prototypes were tested for their ease of navigation and aesthetic before being turned into a final design. For a full description of this process see Morrison et al [12].

### Technology Platform

The app was developed to run on iOS and Android operating systems, and targeted to tablet and phone form factors, though it will also run on a desktop or laptop computer. Due to resource and timing constraints, a decision was made to create a responsive Web-based app that functions similarly to a native app and scales content based on the size of the device’s screen. The app was developed using the Java Enterprise Edition (EE) version 7 stack. The app front-end was created using Cascading Style Sheets (CSS) version 3 and Hypertext Markup Language (HTML) version 5.

### Integration With External Sources

A key requirement that emerged from the design process was the ability to access information from the patient’s EHR in as close to real-time as possible. CCHMC’s EHR vendor is Epic (Epic Systems, United States). Oklahoma is transitioning vendors. Many EHR vendors, including Epic, now offer Web services that can be used to retrieve production data, providing an opportunity for real-time access. As the primary users of this app are patients and families, we focused on those Web services that support Epic’s personal health record, MyChart. Doing so allows us to leverage MyChart’s authentication and authorization protocols, which means parents and families can sign on with their MyChart username and password, obviating the need for a second set of credentials. Our primary Web services of interest were those relating to the retrieval of laboratory results and appointment information.

Content on medical terms and the list of medication names were obtained from databases maintained by the National Cancer Institute's (NCI) Dictionary of Cancer Terms and Drug Dictionary, respectively. The NCI Drug Dictionary was used to retrieve more medication information from services that query Medline Plus, which is run by the National Library of Medicine (NLM). Additional educational materials were obtained from the Children’s Oncology Group (COG) and care teams within the CCHMC’s Cancer and Blood Diseases Institute. These electronic documents reflect much of the same content from the traditional paper binders that are given to families. Links to disease specific information were also incorporated as resources along with information on health promotion and disease prevention.

## Results

**Figure 1:**
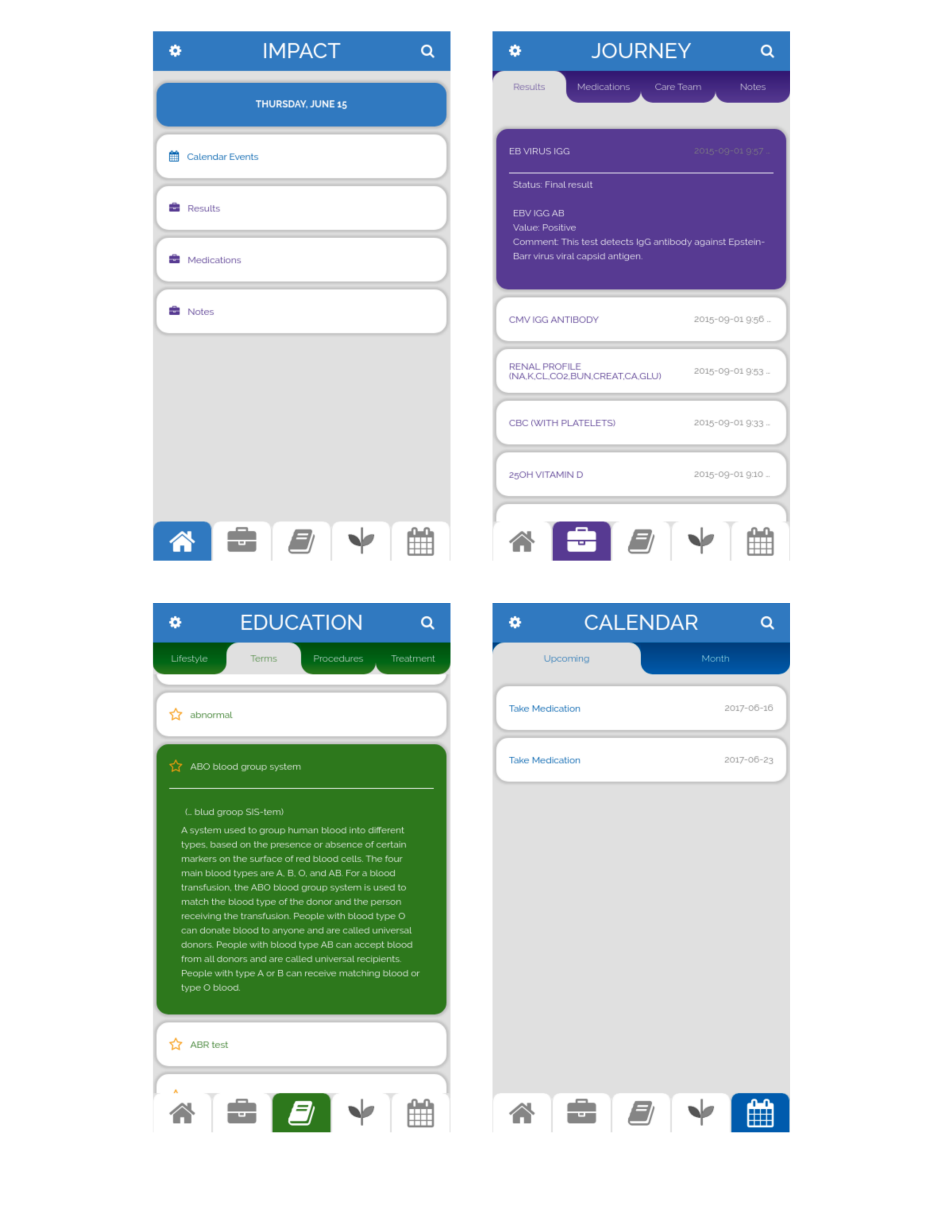
Screenshots of the mobile app. Examples of the home screen (top left), and content from the Journey (top right), Education (bottom left), and Calendar (bottom right) sections. The lab results in the Journey section are simulated. The Study tab is not shown, as the intervention is ongoing.

The ethnographic design process resulted in a framework that divided the content of the mobile app into 4 sections: (1) Journey, which conveys information about the patient’s current treatment and other data from the EHR; (2) Education, where users can find background material and additional medical information; (3) Calendar, which allows users to view upcoming appointments at the medical center or other items that they have added; and (4) Study, the section where participants in the RCT document the study data. The content of each section is detailed in Table 1, along with the expected source of that information. The content of the “Study” tab is not shown as the intervention is still ongoing. Example screens from the application are shown in Figure 1.

**Table 1:** Functionality of the mobile app by section and the expected source of information for each component.

Section	Feature	Description	Source of information
Journey	Results	Provides a list of the patient’s laboratory results and the ability to drill into individual results	EHR^a^
	Medications	A list of the patient’s current medications	User-generated from lists sourced from the NCI^b^ Drug Dictionary and Medline Plus^c^
	Care team	Information on members of the patient’s care team (eg, picture, title, contact information, specialty, etc)	User adds team members using text auto-complete; information pulled from hospital systems
	Notes	Any text notes that the family wishes to document	User-generated
Education	Lifestyle	Background material on topics such as health and wellness, nutrition, and school and learning	Educational handouts from CCHMC^d^ and the COG^e^
	Terms	A list of common medical terms and their definitions	NCI^f^ Cancer Terms database
	Procedures	Descriptions of common procedures used in the treatment of cancer, information on transfusions, and on how to interpret laboratory results	Educational handouts
	Treatments	Background information on the treatment process and other ancillary information related to the care process	Organizational handouts from CCHMC and the COG
Calendar	Upcoming	A description of the patient’s next appointment at the medical center, (including location and date/time) or other event entered by the user	EHR or user-generated
	Month view	Monthly view of appointments pulled from the EHR or manually added by the user	EHR or user-generated

^a^EHR: electronic health record (MyChart Web service).

^b^NCI: National Cancer Institute.

^c^While it is possible to retrieve these data via Web services, there were concerns about the accuracy of this information.

^d^CCHMC: Cincinnati Children’s Hospital Medical Center.

^e^COG: Children's Oncology Group.

^f^NCI: National Cancer Institute.

At the time of final submission (June 2017), the app was being used by 63 participants (patient or family) across the 2 trial sites. There have been approximately 1970 logins since it was deployed, with iOS and Android devices accounting for roughly 75% (66/81) of all device activity (some users log in on multiple devices). As the trial is ongoing, we do not yet have results on the utility of the app. The app is used with families during each IMPACT intervention session, which provides an opportunity to test functionality on a regular basis. Families are also asked to report any problems that occur during their use of the app. These issues are addressed in real-time during the IMPACT sessions. App content, including links to, and content from, external sources, will be reviewed and updated on a yearly basis. We will also monitor usage of the app through Google Analytics, and participants will also be asked for feedback on ease of use, navigation, and overall satisfaction.

### Design Trade-Offs

Informatics staff participated in the ethnographic design process and provided feedback about the feasibility of implementing certain features during the pilot period. This helped ensure that the expectations of the design participants were aligned with what could be delivered given the project budget and development timeline. Examples of features that were removed before the design was finalized included the ability to use the app to chat with or send messages to the care team, to allow provider input of personalized treatment protocols, and real-time notifications about new or updated laboratory results. The app provides the ability for users to quickly find material based on their needs. Information on medications or medical terms is indexed and easily searchable, as are the educational modules, which have been developed to provide guidance on situations faced by families dealing pediatric cancer (eg, returning to school, dealing with siblings). Our original intention was to provide tailored material to each patient/family, but we found that it was not possible to develop individual protocols by treatment course at each stage of treatment within the project timeline. This work is currently underway as part of a separate project, and our intent is to integrate the material in a future version of the app. As an alternative, based on patient and family feedback, we have provided links to trusted sources where some of this information can be found (ie, NCI and COG).

### Integration With External Sources

**Figure 2:**
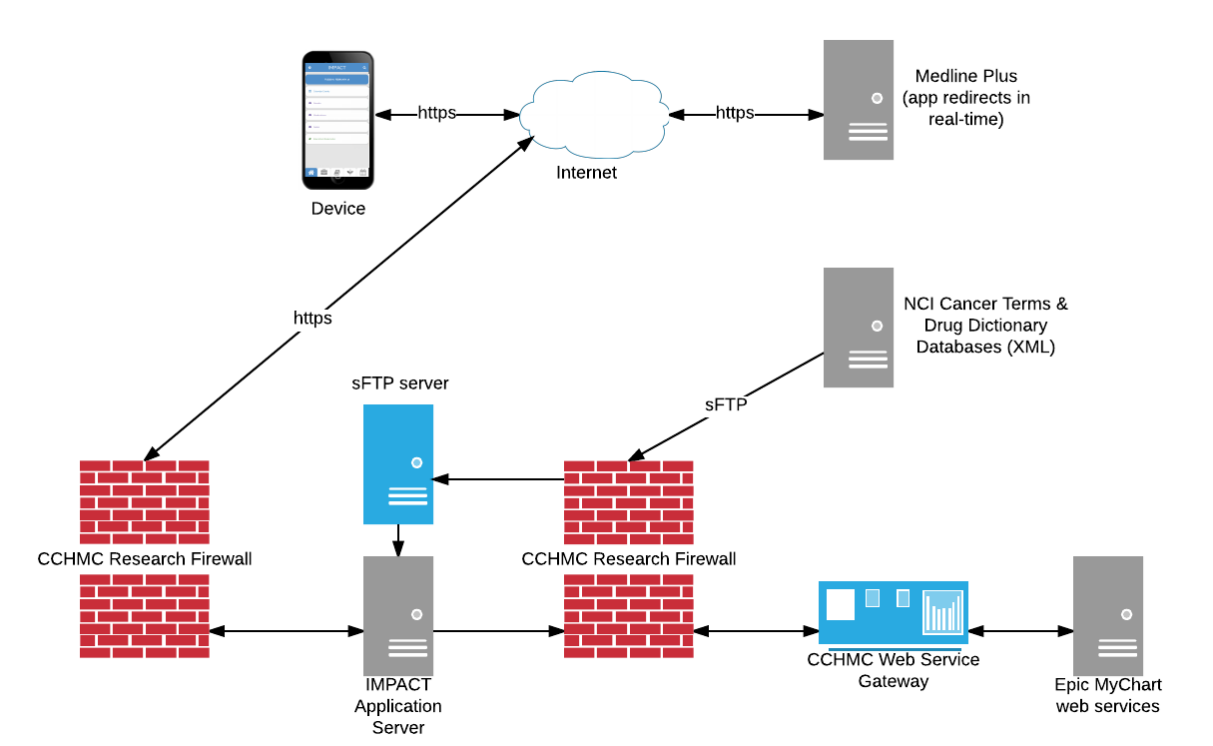
Diagram of the app infrastructure. Calls to Medline Plus are redirected in real-time. Content from the National Cancer Institute (NCI) is staged on the Illness Management and Parental Adjustment to Cancer Treatment (IMPACT) app server. Requests to the Epic Web services are brokered through the Cincinnati Children’s Hospital Medical Center (CCHMC) Web service gateway.

Content from the NCI Dictionary of Cancer Terms and Drug Dictionary were retrieved as Extensible Markup Language (XML) files and staged on the IMPACT app server. A public application program interface (API) that would have allowed us to retrieve this information in real-time was not available at the time of our implementation. The NCI supports a widget that would allow the dictionary to be embedded in a website, but this functionality did not meet our design needs. Following a process recommended by the NCI staff that maintained these databases, we retrieved the content as a series of XML files over the secure File Transfer Protocol (sFTP). We could receive notifications if updates were made to these databases, which would provide an opportunity to download a new version. Integration with the services provided by the NLM was straightforward. Medline Plus is configured to support responsive design and requests were redirected from the app. The material that was sourced from the traditional educational binders was more difficult to incorporate into a seamless mobile experience. Much of this content was directly converted from a text-heavy, paper-handout format, which is difficult to read on a mobile device. A diagram that illustrates the app infrastructure is shown in Figure 2. All traffic to external websites (eg, Medline Plus) is handled as redirects on the user’s device. All data are encrypted in transit via Secure Sockets Layer (SSL) encryption. The app database is currently being migrated so that the data are also encrypted at rest.

Because the Oklahoma site was transitioning EHRs, our integration efforts were focused on the CCHMC EHR. For non-CCHMC users, screens were created within the administrative interface of the app to allow coordinators to enter data manually. Accessing and retrieving data from the CCHMC EHR Web services was successful, but presented several hurdles. The lack of a formal institutional process for requesting Web service access, with documentation of all of the steps needed to for completion proved to be the biggest barrier. This was compounded by the fact that we had difficulty in identifying the most appropriate Web services to use in order to obtain the data in question. Documentation on available services was sparse, making it a challenge to figure out which service was the most appropriate. Finally, in our institution’s development and testing environments, Web services were configured to point at different instances of the EHR. Some services would return old production data, while others would return dummy results. After our project was completed, this was eventually resolved by creating a process that ensured that each time a Web service was activated, it was activated in all environments.

## Discussion

### Lessons Learned

Applying the process of ethnographic design resulted in a specification that can meet the needs of prospective users. Having informatics staff engage in the design process itself can provide benefits as decisions are made on how to translate specifications into an actual product. Trade-offs are often necessary, whether due to budgetary or time constraints, technology limitations, or other factors. Identifying potential challenges early allows information and possible solutions to be factored into the final result or future iterations. For example, the ability for families to interact with their care team through the app was identified as a high priority feature, but we did not want to create a channel that would direct communication to a clinician’s personal mobile device. MyChart services exist that allow external apps like the one we created to utilize “official” communication channels. We ultimately decided that the inclusion of messaging in an initial release would introduce unnecessary complexity, but we were able to outline how the process might work in the future. Real-time notification of new or updated laboratory results is another example of a desired feature that was ultimately removed from the final design. In this case, no default service exists that would trigger an external alert that new or updated information was available. This functionality could be approximated by periodically polling the result services for updates, but we were concerned about the potential stress that this might put on the production EHR, so this feature was also deferred.

To successfully obtain data from the EHR via Web services, we had to overcome a number of hurdles, but these proved to be primarily due to institutional, social, and cultural issues. The lack of a formal process for requesting access to the EHR Web services and ensuring that the request, once approved, would be completed, proved to be the single biggest bottleneck. There were various institutional channels for socializing the project and receiving executive approval, but ensuring that this approval translated into action at the staff level was more difficult. At CCHMC, informatics is a separate unit from hospital information technology (IT), and this project represented one of the first requests for access to EHR Web services from an outside team. As a result, the hand-offs that were necessary to take a request from approval to completion had not been defined, making it difficult to know whether work was in progress or if things had been stalled because a staff member was waiting for additional information. This was resolved by documenting the areas where informatics staff faced difficulty along with suggested solutions, and submitting this to the executive leadership of the hospital IT department. We were then able to collaborate on a more defined process. Once this process was better articulated, frequent communication with the hospital IT staff ensured that continued progress was achieved.

There were several other hurdles faced during the initial implementation that will likely exist at other institutions. First, at CCHMC, like many institutions, there are differences in the underlying EHR environments used for testing and development activities, particularly in whether they were populated with old production data or dummy data. At the time of implementation, the full suite of Web services was not active in all environments. Therefore, we had to use a mix of development and testing services, meaning one service would return real values, while another would return dummy results. This greatly complicated our validation activities. Another challenge was related to the sheer number of Web services available within the EHR. While the EHR contains several hundred native Web services, there are thousands of print groups that can function in the same manner. As a result, there may be hundreds of ways to request a certain type of data. These interfaces have often been created for a specific purpose and apply different filters to the data that are queried, resulting in a unique view of the results. Because of these filters, they can also vary drastically in their performance. Related documentation is sparse, making it difficult to determine which interface to utilize for a given purpose. One potential strategy to address this issue would be for an institution to create a glossary or list of preferred or validated services for a given data type or data domain. This could also be broken down by use case, user base (eg, patient or clinician), or hospital setting (eg, ambulatory versus inpatient) to further streamline the process and shorten development time. Ensuring that the same set of services are also active in every EHR environment (ie, development, testing and production) would also help foster success.

### Conclusions

We were able to successfully deploy a mobile app with a custom user interface that can integrate with the EHR to retrieve laboratory results and appointment information using vendor-provided Web services. We used the MyChart services for authentication and authorization, allowing families to utilize their same usernames and passwords to log in. The use of ethnographic design provides an opportunity for researchers to work with stakeholders to identify and develop new interfaces or methods of interacting with the EHR that can serve needs that are not being met with current approaches. It is possible to do this using technology that exists at most institutions. The ethnographic design process itself is best served when informatics development staff are engaged along with the interviewees. Informatics staff can be upfront about any potential challenges in requesting a certain feature, which can be worked into the design and help manage expectations. Developers should expect to face hurdles when integrating with the EHR, but many of them can be addressed with frequent communication and thorough documentation. Continued executive sponsorship is also a key factor, especially the first few times these projects are attempted. In the end, a repeatable process should be achievable, allowing the development of such apps to occur more frequently.

## Abbreviations

CCHMC: Cincinnati Children’s Hospital Medical Center
COG: Children’s Oncology Group
EHR: electronic health record
ESO: education and support only
IMPACT: Illness Management and Parental Adjustment to Cancer Treatment
IT: information technology
NCI: National Cancer Institute
NLM: National Library of Medicine
OUHSC: University of Oklahoma Health Sciences Center
RCT: randomized controlled trial
XML: Extensible Markup Language
